# Influence of Forced Aeration and Season on Food Waste Composting: Organic Compound Degradation and Compost Quality

**DOI:** 10.1021/acsomega.5c10007

**Published:** 2026-01-26

**Authors:** Ranielle Nogueira da Silva Vilela, Juliana Dias de Oliveira, Érika do Carmo Ota, Marco Antonio Previdelli Orrico Junior, Brenda Kelly Viana Leite, Tarcila Souza de Castro Silva, Luís Antonio Kioshi Aoki Inoue, Ana Carolina Amorim Orrico

**Affiliations:** † Department of Animal Science, College of Agricultural Sciences, 186079 Federal University of Greater Dourados, PO Box 364, Dourados, MS 79.804-970, Brazil; ‡ 611308 Brazilian Agricultural Research Corporation (Embrapa Agropecuária Oeste), Dourados, MS 79.804-970, Brazil

## Abstract

Food waste composting is essential for improving its sustainable management, providing effective solutions for its recycling, and mitigating the environmental impacts of improper disposal. This study aimed to investigate the need for forced aeration in static piles during the composting of food waste, both in winter and in summer. Samples were collected on days 50, 70, and 90 of composting to analyze the degradation of organic constituents and the quality of the final compost. In the summer, aeration favored the degradation of ether extract (EE) at 50 days (78.2%) and nitrogen (N) at 70 days (86.1%) and promoted the greatest reduction of total solids (TS, 73.9%), carbon (C, 75.1%), N (70.2%), and EE (97.4%) by the end of the composting process. In winter, although aeration promoted degradation at 50 days (TS, N, and EE) and 70 days (N, EE), reductions at 90 days were more pronounced for TS (73.3%) and C (75.9%) in nonaerated windrows or similar for N (70.1%) and EE (96.0%). Composting was more efficient in nutrient release and formation of humic acid during the summer. Thus, we can conclude that food waste composting, with or without aeration, effectively recycles organic waste, promoting degradation and nutrient-rich compost production, with seasonal variations influencing the process.

## Graphical abstract

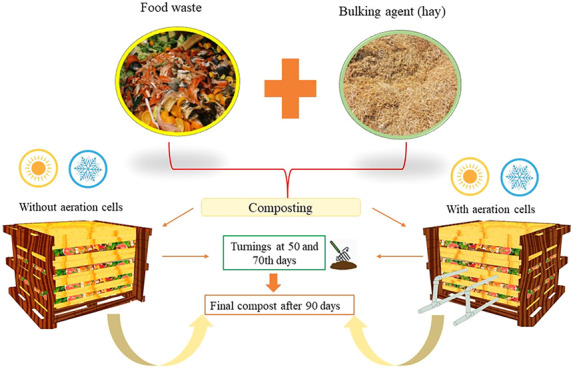

## 1 Introduction

Food waste is generated daily worldwide from households, restaurants, and food processing industries. It is estimated that 1.3 billion tons of food waste are generated annually, representing one-third of all food produced for human consumption.^1^ This waste poses several threats to public health and the environment due to greenhouse gas emissions, pathogenic microorganisms, and diseases transmitted by vectors.^2^ Proper management of this waste has become one of the main challenges today, as many nations have prohibited its use in animal feed production.

Landfills are a common method for food waste disposal; however, in some countries, this technique has been banned due to soil contamination. Anaerobic digestion is an ecological technique for treating food waste,^3^ but its widespread application is hindered by technical and economic obstacles, such as the accumulation of volatile fatty acids, foam formation, and high financial costs.^4^

Given the limitations of food waste management methods, composting emerges as an effective alternative for treating and recycling nutrients present in this waste. Composting is a well-established and widely adopted technique due to its ease of operation and its ability to produce high-quality organic fertilizer.^5^

Considering the contaminant load of food waste, it is recommended to compost in static windrows with no handling of the material for at least 50 days after the process begins. The main challenge for this composting is to maintain aeration conditions throughout the entire pile, as the lack of oxygen during the process slows the degradation of organic constituents and favors the proliferation of flies and foul smells. The bulking agent must provide adequate support for the composting material, presenting porous properties sufficient to allow air passage into the pile, prevent leachate formation, and provide a carbon source that adjusts the C/N ratio of the material. In addition, the proportion of the bulking agent to waste must also be adequate. As reported in a previous study,^6^ food waste should be composted with at least 40% bulking agent to ensure efficient decomposition.

The distribution of air throughout the pile’s profile does not depend solely on the bulking agent, as the material in static windrows tends to compact over time due to mass reduction as the material is degraded.^7^ To ensure the oxygen input required for the success of composting, forced aeration must be applied homogeneously throughout the composting cell,^8^ playing a key role in maintaining thermophilic conditions, especially during the active decomposition of organic matter.

Nonetheless, caution is required regarding the aeration rate, particularly considering the time of year in which the composting occurs, as insufficient or excessive aeration can result in immature compost and lead to greater nitrogen loss.^9^ Vilela et al.^10^ used forced aeration at a rate of 0.57 L min^–1^ kg^–1^ OM (organic matter) during the winter and summer seasons. The authors reported that this aeration rate delayed the degradation of the material, particularly in the winter. On the other hand, Wang et al.^11^ used different aeration rates (0.44, 3.25, 6.50, and 11.65 L kg min ^–1^ kg^–1^ dry matter (DM)) during the composting of food waste. The authors concluded that 3.25 L kg min ^–1^ kg^–1^ DM was the most appropriate rate for nitrogen conservation and excellent compost maturity; however, this rate is recommended for composting in closed reactors.

This study differs from previous research by simultaneously evaluating the combined effects of seasonal conditions and aeration on food waste composting. Few studies have examined how seasonal variation in air temperature and humidity interacts with aeration to influence thermal behavior, degradation patterns, and humification dynamics. Our work fills this gap by providing an integrated assessment of physicochemical transformations and compost quality under contrasting environmental conditions.

Therefore, this study was conducted to evaluate the influence of forced aeration and seasons (summer and winter) on the performance of the composting process and on the quality of the final compost.

## 2 Materials and Methods

The research was carried out in the Experimental Area and Laboratory of Agricultural Waste Management, both belonging to the Faculty of Agricultural Sciences of the Federal University of Grande Dourados, located in the city of Dourados, MS, Brazil (22°11′38″ S, 54°55′49′′ W and altitude of 462 m). According to the Köppen classification, the climate of the region is CWA-Humid mesothermal, with hot and rainy summers and dry winters.

To experiment, a completely randomized design was adopted, in a 2 × 2 factorial scheme, represented by aeration (with and without) and carried out in two seasons (winter and summer), with a plot subdivided by times (50, 70, and 90 days) and with two replicates (windrows) per treatment. The waste used in this study was obtained from the discards generated in a university restaurant located in Dourados, MS. This material was collected after lunchtime and contained leftover food that was not consumed by students. The bulking agent material was low-quality Piatã grass hay, crushed into particles of approximately 2.0 cm and associated with food waste at a ratio of 1:3 (mass:mass). This proportion was adopted according to the recommendations of Leite et al.^12^ to avoid the formation of leachate and allow a better C/N ratio in the material at the beginning of composting. Table 1 presents the chemical compositions of the waste and experimental composting piles in the winter and summer.

**Table 1:** Chemical Composition of the Raw Materials and Experimental Treatments Used in the Composting of Food Waste in Static Piles Conducted in the Winter and Summer^a^

	raw material				
		food waste		experimental treatments	
parameter	bulking agent	winter	summer	winter	summer
pH	7.02	4.31	4.45	4.42	4.45
TS (%)	90.00	33.33	28.41	43.84	39.23
VS (% de TS)	93.97	93.80	93.80	94.67	93.31
C (% de TS)	52.36			46.06	42.75
N (% de TS)	0.47			3.93	4.28
C:N	111.40			11.72	9.99
EE (% de TS)	0.69			13.75	5.57
NDF (% de TS)	79.54			52.33	51.13
ADF (% de TS)	49.48			15.49	18.73
lignin (% de TS)	4.95				

a TS: total solids; VS: volatile solids; C: carbon; N: nitrogen; C:N: carbon:nitrogen ratio; EE: ether extract; NDF: neutral detergent fiber; ADF: acid detergent fiber.

The composting cells were built of wood cubes with spacing between the planks, allowing the natural circulation of air inside. The measurement of each cell was 1.2 m × 0.50 m × 1.20 m (L × W × H). The estimated capacity of each windrow was 200 kg of raw material, including organic waste and absorbent material. Each composting cell was internally layered with sombrite to avoid the loss of the material by the spacing of the wood.

The cells were assembled in alternating layers of materials, using the described ratio of organic waste and absorbent material, with one layer of hay and one of food waste, and so, this order was followed until the height of the cell was reached, with the last layer being the bulking agent (Figure 1). Each layer had an average thickness of around 10 cm, which enhanced the contact between the bulking agent and the waste, thereby promoting aeration and creating a more homogeneous decomposition environment throughout the windrow profile.

**Figure 1:**
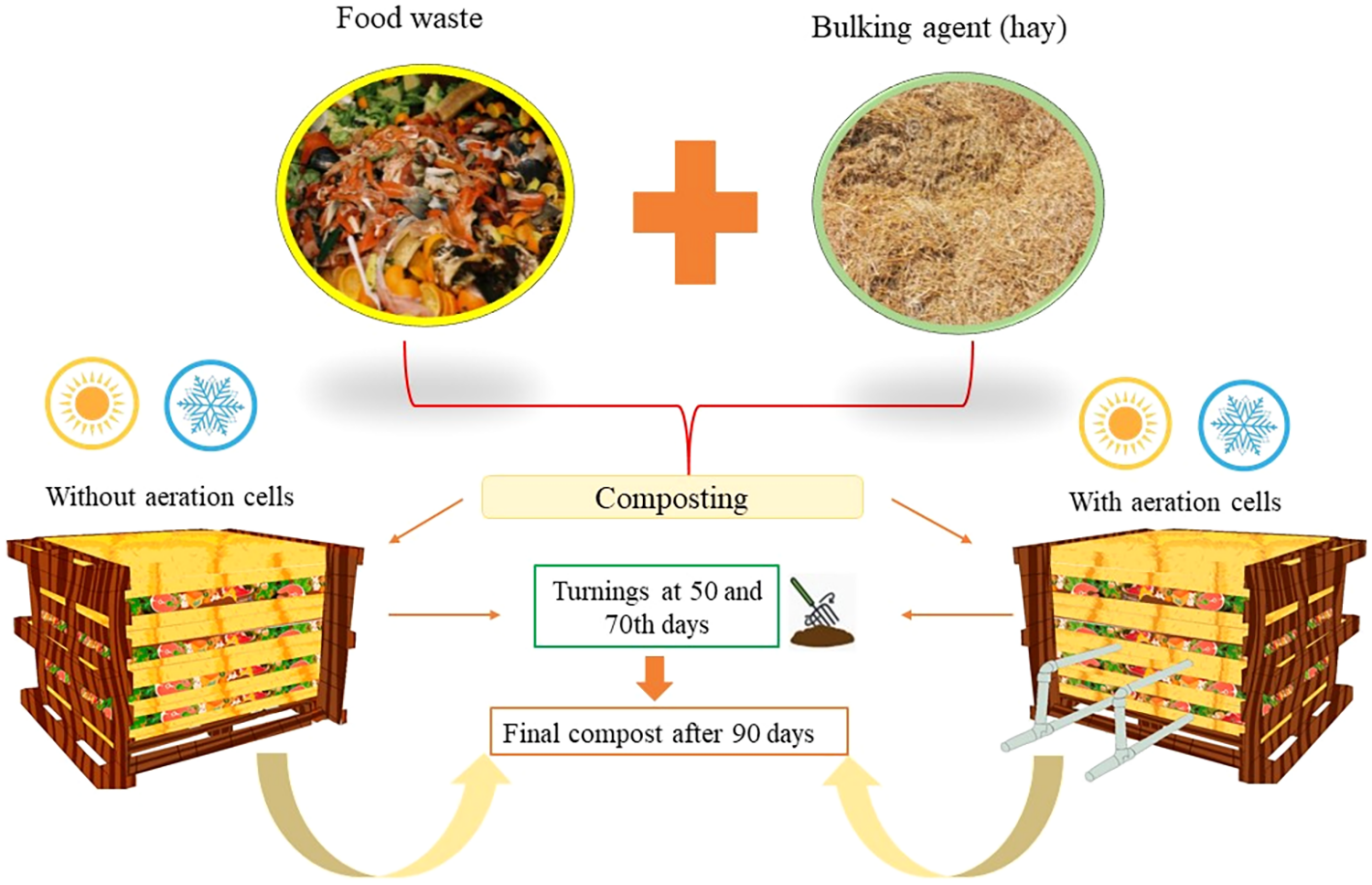
Schematic representation of the experiment.

The cells received aeration through PVC tubes with a diameter of 50 mm that were inserted between the layers of waste, and these pipes were perforated along their lengths so that they could aerate throughout the windrow profile. These pipes were distributed horizontally, throughout the depth of the windrow, with a distance of 25 cm from the base to the first pipe and 55 cm from the base to the second pipe. The pipes were coupled to an air blower motor, which provided a continuous daily flow of 0.57 L kg^–1^ VS min^–1^ as recommended in ref 13.

The total composting period was 90 days, with the first compost turning 50 days and the second turning 70 days of the period. During the compost turning, all of the material inside the cell was removed and placed on a plastic tarp to homogenize and adjust the moisture content; then, the material was returned to the composting cell. The temperature inside each windrow was measured daily with a skewer thermometer at 10 different points that were randomly distributed among the base, center, and top of the windrow to determine the average temperature.

Samples of random points were collected during the turning of the compost to evaluate the degradation of the organic constituents and the quality of the compost. The initial samples were dried by lyophilization due to the high fat content. The samples collected at 50, 70, and 90 days of composting were dried in a forced-air oven for 72 h at a temperature of 60 °C. During the 90 days of composting, the moisture conditions of the windrows were evaluated, randomly selecting points for the collection of samples in the profile and determining the TS so that small amounts of water were added (thus avoiding the formation of leachate) to maintain moisture within the range of 40%–60%. Composting was considered complete when the temperature of the windrows remained stable, the degradation of solids stabilized, and the C levels maintained constant concentrations; then, the windrows were weighed, homogenized, and sampled for the final characterization of the compost.

In the initial material, at 50, 70, and 90 days of composting, the TS, VS, carbon, nitrogen, neutral detergent fiber (NDF), acid detergent fiber (ADF), and lignin were determined. The quality of the compost was determined based on the samples at 90 days of composting when the levels of macrominerals (P, K, Ca, Mg, S, and Na), microminerals (Mn, Fe, Cu, and B), humic acids (HA), and fulvic acid (FAs), and the HA:FA ratio were measured.

The TS and VS contents were measured according to the methodology described in ref 14, and the material pH was determined using the method described by Brazilian Normative Instruction 17/2007.^15^ The NDF and lignin contents were determined according to the methodology described in ref 16 using ANKOM equipment. The concentrations of carbon and nitrogen were determined using the elemental analyzer VARIO MACRO. The levels of HA and FA were determined using the elemental analyzer VARIO TOC, according to the methodology in ref 17. For mineral analysis, the compost samples were submitted to digestion with nitric–perchloric acid following the recommendations of the AOAC^16,18^ . The levels of minerals were determined using an inductively coupled plasma atomic emission spectrometer (ICP–AES), PerkinElmer, model Optima 8300 (Dual View).

To evaluate the influence of season, aeration, and composting time on the degradation of organic constituents and N losses, factors were analyzed independently if the interaction between them was not significant by ANOVA; otherwise, the interactions were unfolded. For the qualitative factors (aeration and season), the means were compared using the Tukey test (*p* < 0.05). For the quantitative factor (composting time), polynomial regression analysis was performed.

To analyze the results of the chemical composition of the final compost (at 90 days), when there was significant interaction by ANOVA, unfolding was performed, considering the season within each aeration level and the aeration within each season, using Tukey’s test to compare the means. When the interaction was not significant, the factors were independently analyzed by Tukey’s test. All analyses were performed using R software (2020).

## 3 Results and Discussion

The temperature in the static windrows remained in the mesophilic range (below 45 °C) during most of the food waste composting period, regardless of aeration or season (Figure 2a,b). In winter, the aerated windrows reached thermophilic temperatures for 24 days, while the nonaerated windrows maintained this phase for only 13 days. During the summer, the aerated windrows reached thermophilic conditions for 19 days, while the nonaerated windrows retained this phase for 29 days. Temperature peaks were observed immediately after the formation of the composting windrows (Figure 2a,b), with values of 55.6 and 43.9 °C for the aerated and nonaerated windrows, respectively, during winter and 52.2 and 50.9 °C during summer. Furthermore, additional temperature peaks were recorded after 50 and 70 days of turning in both conditions.

**Figure 2:**
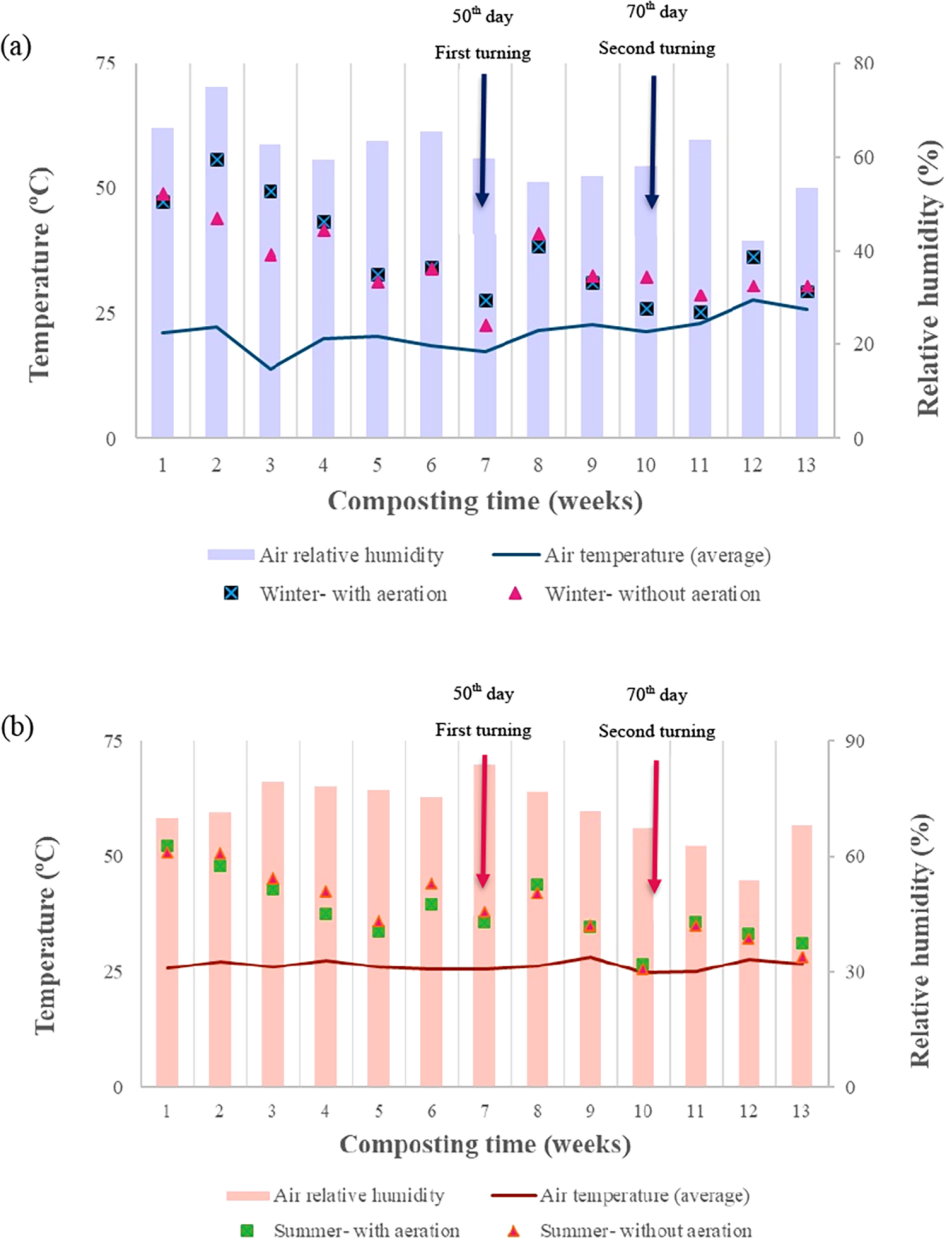
Average weekly air temperature, windrow internal temperature, and relative humidity during the composting of food waste in static windrows with and without aeration in winter (a) and summer (b). All variables represent the mean of three replicates (*n* = 3), and windrow internal temperature values correspond to the daily mean from 10 measurement points.

In addition to the internal temperature dynamics, Figure 2 shows that air temperature and relative humidity differed markedly between seasons, with winter presenting lower ambient temperature (21.1 °C) and lower humidity (59.9%), while summer was characterized by higher air temperature (26.6 °C) and higher relative humidity (72.0%). In Brazil, especially in the central-west region, seasonal contrasts are strongly influenced by humidity, with very dry conditions during the winter and much higher humidity in the summer. This contrast directly affects compost moisture loss from windrows: during dry winter conditions, piles tend to lose water more rapidly, limiting microbial activity and reducing heat retention, a challenge that is intensified under forced aeration. Thus, although “season” was used as the categorical factor in the experimental design, the differences observed between winter and summer clearly reflect the underlying environmental drivers, temperature, and especially humidity, which modulated microbial activity, thermophilic duration, and degradation rates.

Temperature peaks indicate the activity of the decomposer microorganisms. Initially, the waste remains largely intact, with a high concentration of organic matter readily available for degradation.^19^ Over time, despite forced aeration, the material tends to become compacted, which restricts oxygen flow and hinders microbial activity.^20^ The turnings at 50 and 70 days likely restored aerobic conditions by renewing oxygenation and reducing compaction, thereby creating a favorable environment for the development of aerobic decomposer microorganisms. This response explains the additional thermophilic peaks observed during the process and highlights the importance of turning in maintaining composting efficiency, as also reported by other authors.^21,22^

Following the turning performed at day 50, temperatures remained above 45 °C for up to six consecutive days, both in winter and summer, indicating sustained microbial activity and emphasizing the importance of thermal dynamics and moisture in regulating microbial processes.^20^ The limited persistence of the thermophilic phase, observed across all experimental conditions, may be attributed to the heterogeneity of the food waste, which included fruits and vegetables, resulting in an initially acidic pH (Table 1) that hindered microbial colonization. In addition, part of the waste had been previously cooked, which altered its chemical composition by reducing structural components and nutrient availability,^23^ thereby potentially limiting sustained microbial activity. However, pH increased significantly, from 4.42 to 7.81 in winter and from 4.45 to 7.80 in summer, indicating active microbial metabolism, primarily due to degradation of nitrogenous compounds and ammonia release.^24^

Aeration, seasonality, and composting time significantly influenced the degradation of TS, C, N, and EE (*p* < 0.05; Table 2 and Figures 3, 4, 5, and 6). As expected, reductions in all constituents increased with the composting time. The total reductions in TS, C, N, EE, and NDF after 90 days of composting were similar in both winter and summer in the absence of aeration and for N under aerated conditions, highlighting the effectiveness of organic matter degradation regardless of the season. However, the results for TS, C, EE, and NDF differed when aeration was applied (*p* < 0.01; Table 2).

**Figure 3:**
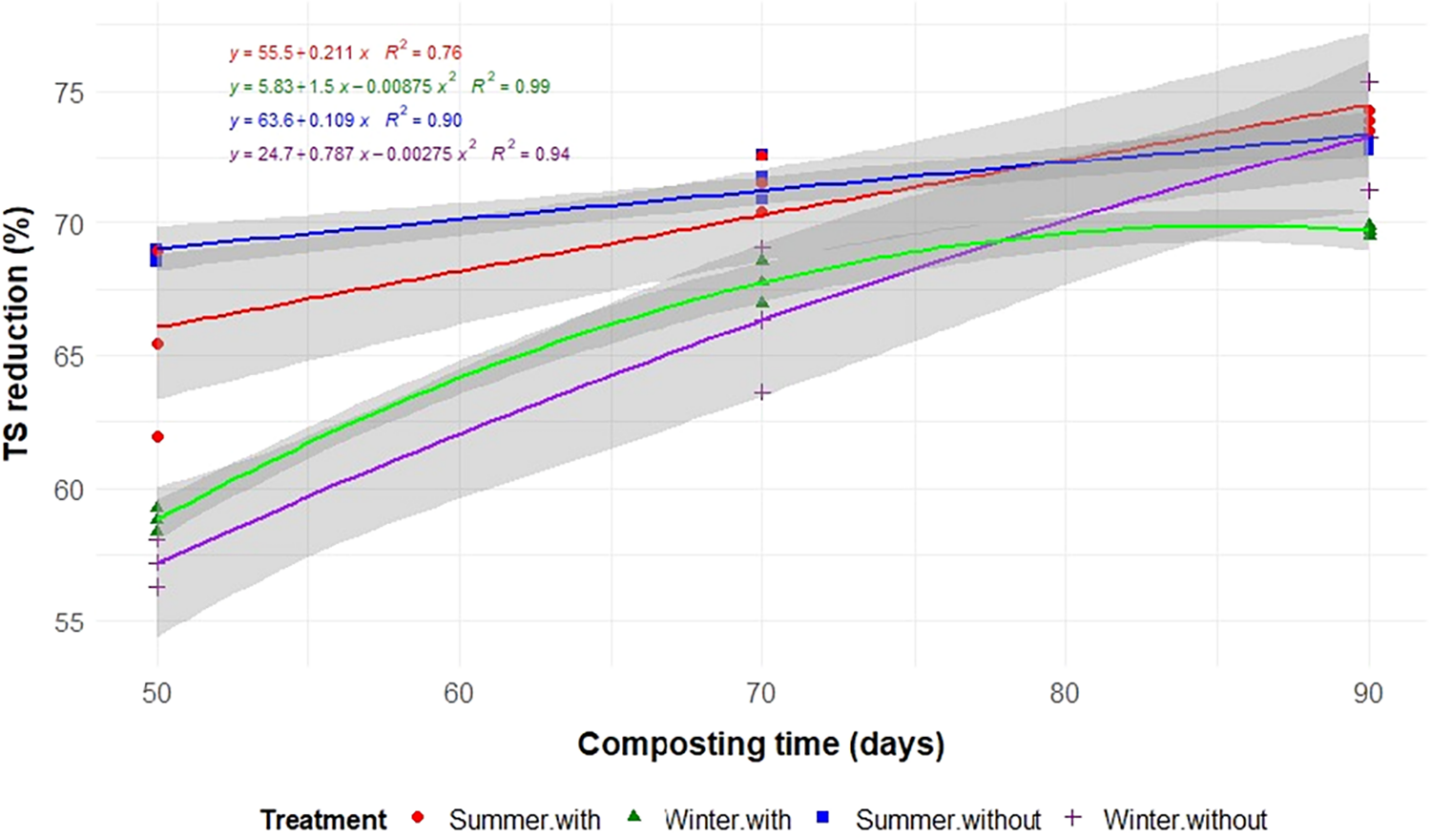
Reductions of total solids (TS) during the composting of food waste in static cells, with or without forced aeration, and conducted in the summer and winter.

**Figure 4:**
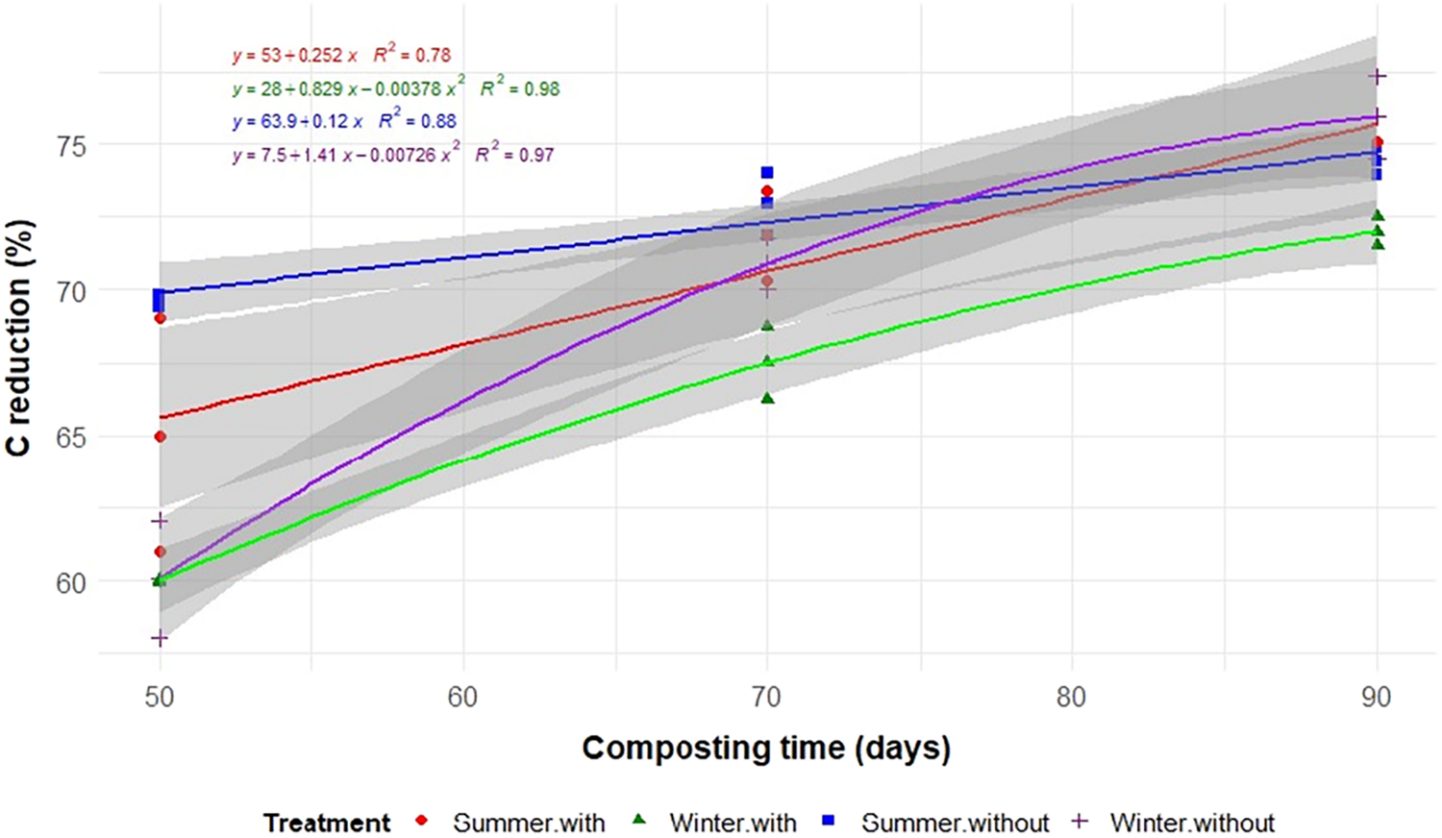
Carbon reductions (C) during the composting of food waste in static cells, with or without forced aeration, and conducted in the summer and winter.

**Figure 5:**
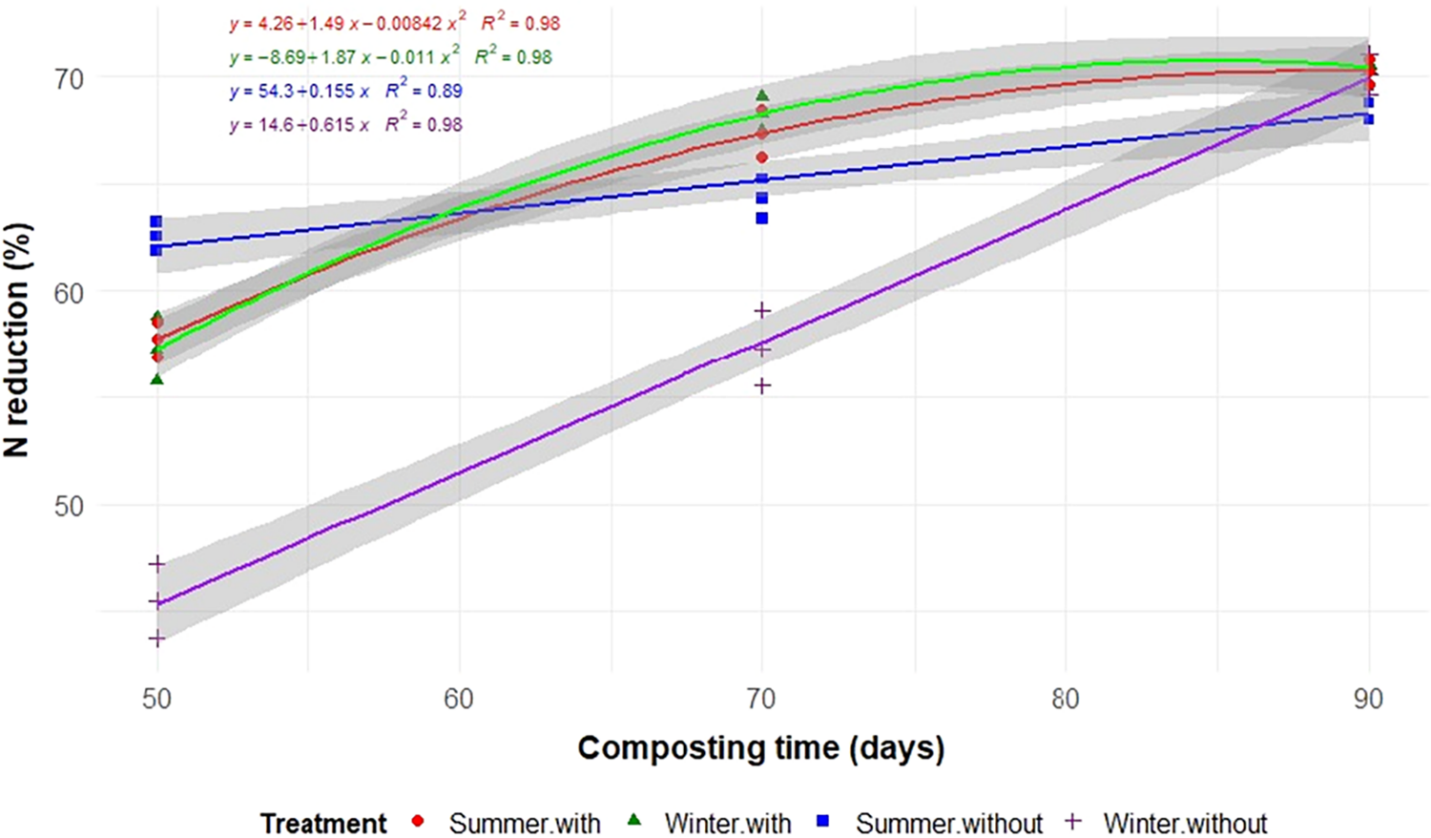
N reductions during the composting of food waste in static cells, with or without forced aeration, and conducted in the summer and winter.

**Figure 6:**
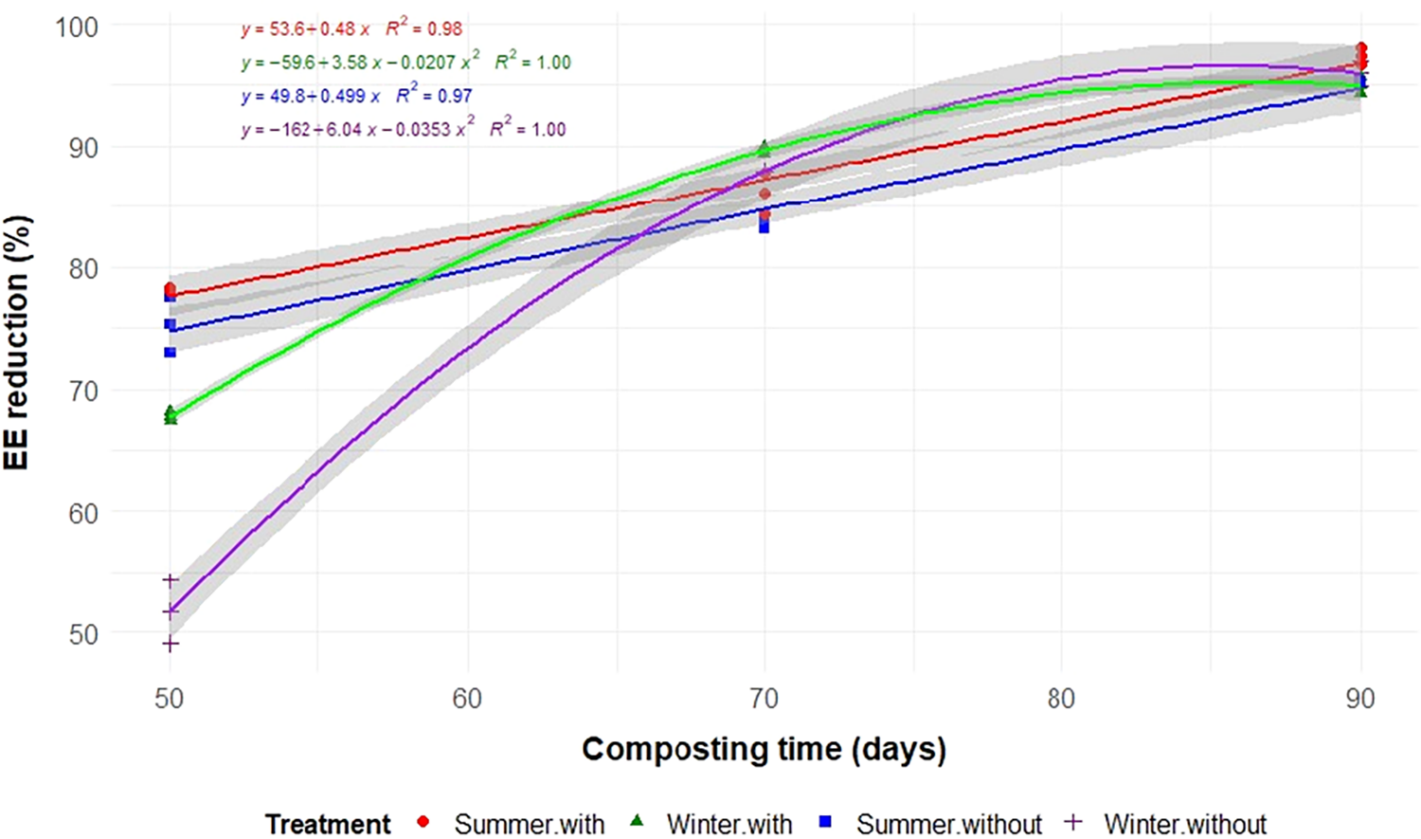
Reductions of ether extract (EE) during the composting of food waste in static cells, with or without forced aeration, and conducted in the summer and winter.

**Table 2:** Reductions of Total Solids (TS), Carbon (C), Nitrogen (N), Ether Extract (EE), Neutral Detergent Fiber (NDF), and Acid Detergent Fiber (ADF) during Composting of Food Wastes, in Static Windrows, Conducted in Summer and Winter, with or without Aeration at 50, 70, and 90 Days of Composting^a^

		composting time (T)									*p*-value						
season (S)	aeration (A)	50			70			90			S	A	T	S × A	S × T	A × T	S × A × T
TS Reduction (%)																	
winter	with	58,8	b	(X)	67,7	b	(X)	69,7	b	(Y)							
	without	57,2	y	(Y)	66,3	y	(X)	73,3	x	(X)							
											***	NS	***	NS	***	NS	**
summer	with	65,5	a	(A)	71,5	a	(A)	73,9	a	(A)							
	without	68,8	x	(A)	71,7	x	(A)	73,1	x	(B)							
C Reduction (%)																	
winter	with	60,0	b	(X)	67,5	b	(Y)	72,0	b	(Y)							
	without	60,0	y	(X)	70,9	y	(X)	75,9	x	(X)							
											***	***	***	NS	***	NS	**
summer	with	65,0	a	(B)	71,8	a	(B)	75,1	a	(A)							
	without	69,6	x	(A)	72,9	x	(A)	74,4	x	(B)							
N Reduction (%)																	
winter	with	57,3	a	(X)	68,3	a	(X)	70,4	a	(X)							
	without	45,5	y	(Y)	57,3	y	(Y)	70,1	x	(X)							
											***	***	***	***	***	***	***
summer	with	57,7	a	(B)	67,4	a	(A)	70,2	a	(A)							
	without	62,6	x	(A)	64,3	x	(B)	68,8	x	(B)							
EE Reduction (%)																	
winter	with	67,7	b	(X)	89,7	a	(X)	95,0	b	(X)							
	without	51,7	y	(Y)	87,9	x	(Y)	96,0	x	(X)							
											***	***	***	***	***	***	***
summer	with	78,2	a	(A)	86,1	b	(A)	97,4	a	(A)							
	without	75,3	x	(B)	83,8	y	(A)	95,2	x	(B)							
NDF Reduction (%)																	
winter	with	38,5	(b)	(Y)	47,0	(b)	(Y)	55,0	(b)	(X)							
	without	41,4	(y)	(X)	51,6	(y)	(X)	57,9	(x)	(X)							
											***	***	***	NS	**	*	*
summer	with	44,0	(a)	(B)	53,7	(a)	(B)	59,3	(a)	(A)							
	without	51,5	(x)	(A)	57,0	(x)	(A)	59,0	(x)	(A)							
ADF Reduction (%)																	
winter		33,8	b		42,7	b		49,1		b	***	**	***	NS	*	NS	NS
summer		46,6	a		52,5	a		56,8		a							

a Letters (a) and (b), in the column, compare season effect, with aeration and within each specific composting time. Letters x and y, in the column, compare season effect, without aeration and within each specific composting time. Letters (A) and (B), in the column, compare the effect of aeration, in the summer season and within each specific composting time. Letters (X) and (Y), in the column, compare the effect of aeration, in the winter season and within each specific composting time. Means followed by different letters differ from each other by the Tukey test (***: *p* < 0.001, **: *p* < 0.01, *: *p* < 0.05, and NS: not significant).

In summer, aeration significantly accelerated the degradation of TS, C, N, and EE, with reductions of 73.9, 75.1, 70.2, and 97.4%, respectively, by 90 days. In contrast, during the winter, the absence of aeration resulted in greater reductions in TS and C, with nonaerated windrows showing higher reductions (73.3% for TS and 75.9% for C) compared to aerated piles (69.7% for TS and 72.0% for C). The decomposition rates align with these results, as shown by the regression (Figures 3–6). In winter with aeration, the TS reduction increases initially but stabilizes and even decreases after a certain point, following an inverted parabola pattern (Figure 3).

A more pronounced degradation of NDF was observed during the summer compared to the winter, and under nonaerated compared to aerated conditions (Table 2 and Figure 7). The greater degradation of NDF under summer and nonaerated conditions may reflect environmental conditions more favorable to fungal activity, which is known to play a key role in the breakdown of fibrous constituents.^22,25^ Although fungal development was not directly measured in this study, the observed patterns are consistent with literature reports describing enhanced fungal contribution under warmer and more humid composting conditions. In the composting system, cellulose degradation is more effectively carried out by fungi than by bacteria. This predominance becomes particularly evident when the substrate exhibits a high degree of lignification, which increases the reliance on fungal activity for efficient biodegradation.^26^ Regarding ADF, degradation was greater (*p* < 0.05) throughout the summer composting period (56.8%) compared to winter (49.1%).

**Figure 7:**
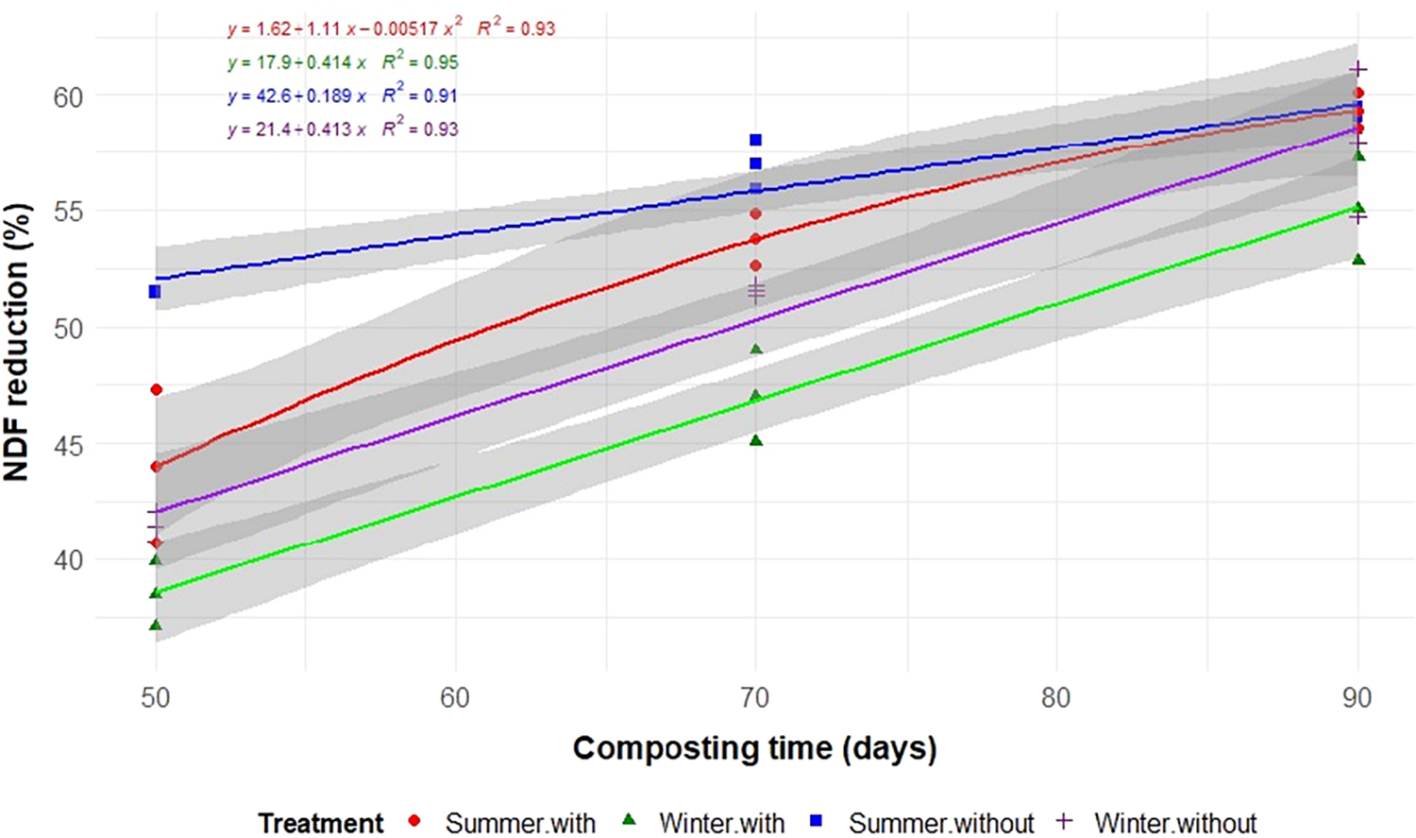
Reduction of neutral detergent fiber (NDF) during the composting of food waste in static cells, with or without forced aeration, and conducted in the summer and winter.

The final concentration of N ranged from 24.2 to 31.0 g kg^–1^ of compost (Table 3), consistent with those reported by Xiong et al.^21^ who observed concentrations between 26.17 and 31.70 g kg^–1^ of compost, with reductions ranging from 32.8% to 49.2%. Similar results were found by Orrico et al.^27^ in fish waste composting, with N reductions ranging from 55.03% to 79.88%, and concentrations between 27.12 and 30.42 g kg^–1^. Vilela et al.^28^ reported reductions from 32.04% to 63.4% at the end of ruminant waste composting, with N concentrations between 28.8 and 36 g kg^–1^ of compost. The intensive mineralization of organic nitrogen to ammonium ions under high-temperature conditions favors ammonia volatilization,^29^ which is the main factor responsible for nitrogen loss during composting. Furthermore, the nitrification process tends to increase gradually in the final stages of composting due to reduced substrate moisture, improved aeration conditions, and lower temperatures.^30^

**Table 3:** Macro- and Micronutrient Composition in Compost Resulting from Food Waste Composting in Static Piles, with or without Aeration, and Conducted during the Summer and Winter Seasons

season (S)	winter		summer		*p*-value		
aeration (A)	without	with	without	with	S	A	S*A
N (g kg^–1^)	24.9 ± 0.9	24.2 ± 2.8	29.7 ± 0.5	31.0 ± 0.6	***	NS	NS
P (g kg^–1^)	6.4 ± 1.9	5.1 ± 0.8	6.8 ± 0.6	7.0 ± 0.1	NS	NS	NS
K (g kg^–1^)	12.8 ± 0.5	15.1 ± 1.2	27.6 ± 0.4	28.2 ± 1.0	***	*	NS
Ca (g kg^–1^)	5.4 ± 0.5	5.9 ± 0.8	9.0 ± 0.1	6.8 ± 0.4	***	*	**
Mg (g kg^–1^)	2.9 ± 0.1	3.3 ± 0.2	5.3 ± 0.2	4.7 ± 0.3	***	NS	**
S (g kg^–1^)	3.6 ± 0.5	3.9 ± 0.4	6.5 ± 0.3	6.1 ± 0.2	***	NS	NS
Zn (mg kg^–1^)	58.1 ± 0.7	73.4 ± 7.6	85.7 ± 1.4	86.1 ± 1.8	***	***	***
Mn (mg kg^–1^)	181.7 ± 14.2	216.5 ± 5.5	399.9 ± 0.9	358.5 ± 7.1	***	NS	***
Fe (mg kg^–1^)	1124.1 ± 25.7	755.3 ± 29.3	2854.4 ± 75.1	2910.5 ± 39.6	***	***	***
Cu (mg kg^–1^)	7.6 ± 0.2	8.8 ± 0.4	24.3 ± 1.6	24.2 ± 0.4	***	NS	NS
B (mg kg^–1^)	9.4 ± 1.1	9.6 ± 0.6	23.0 ± 1.3	22.3 ± 0.7	***	NS	NS
Na (mg kg^–1^)	12.9 ± 1.5	14.0 ± 0	14.3 ± 0.0	15.3 ± 0.5	*	NS	NS
humic acid (HA, mg g^–1^)	275.1 ± 9.2	251.7 ± 0.8	271.2 ± 6.7	260.0 ± 8.5	NS	**	NS
fulvic acid (FA, mg g^–1^)	19.5 ± 0.2	20.1 ± 0.4	18.4 ± 0.3	19.2 ± 0.7	**	*	NS
HA/FA	14.1 ± 0.3	12.5 ± 0.3	14.7 ± 0.1	13.5 ± 0.1	***	***	NS

In contrast, nonaerated windrows in the winter showed greater retention of organic carbon, which could be beneficial for retaining carbon in the compost.^29^ EE reduction is crucial for improving the compost quality, as it indicates the ability of the system to degrade lipids. Initially, EE content was high, but during the first 50 days of composting, EE reduction exceeded 50%, and by the end of the process, a total decrease of over 90% was observed (Figure 6). The moisture likely facilitated the activity of lipolytic microorganisms, enabling efficient degradation.^31^

Phosphorus concentration was not influenced (*p* < 0.05) by either season or aeration (Table 3), aligning with findings reported in refs 13,22 . Among the primary macronutrients essential for soil fertilization (N, P, and K), N and K exhibited higher concentrations (*p* < 0.01) during summer composting under forced aeration reaching 31.0 and 28.2 g kg^–1^, respectively. In contrast, the *P* levels remained comparatively stable at 7.0 g kg^–1^. This increase is likely associated with the enhanced microbial activity promoted by aeration, which accelerates the decomposition of organic matter and facilitates the release of nutrients such as N and K into compost. This effect was markedly pronounced during the summer, presumably due to higher environmental temperatures that further stimulate microbial process and nutrient mineralization.

The concentrations of S, Cu, B, and Na were influenced by seasonal variation but not by aeration, exhibiting higher levels (*p* < 0.001) during the summer. In contrast, the concentrations of K, Zn, Fe, Ca, Mg, and Mn were affected by both season and aeration. The highest concentrations (*p* < 0.001) were recorded in the summer, specifically under forced aeration for K, Zn, and Fe and under passive conditions for Ca, Mg, and Mn.

The concentration of HA (Table 3) was significantly influenced by aeration (*p* < 0.01), with higher levels observed in compost produced under nonaerated conditions. The formation of humic acids results from the degradation of lignin and marks the most advanced stage of compost humification. According to Yao et al.,^32^ reduced internal temperature fluctuations during the composting process facilitated a more complete biodegradation of lignin, thereby promoting a greater accumulation of HA. The generation of FA and the HA/FA ratio (Table 3) were influenced by both season and aeration (*p* < 0.0001). The greatest accumulation of FA (19.8 mg g^–1^ of compost) was attained during the winter under forced aeration, whereas the highest HA/FA was achieved in the summer under nonaerated conditions. Carboxylic, phenolic, and amino groups constitute the predominant functional groups in humic substances, with carboxylic and phenolic groups serving as the principal contributors to total acidity.^33^ Although HA represents a more advanced stage of humification, with carboxylic and phenolic groups bound to high molecular weight and stable structures, FAs are less stabilized, lower molecular weight compounds, thus exhibiting greater total acidity.^34^

High molecular weight humic acids (HAs) is characterized by higher concentrations of aromatic carbon, while low molecular weight fulvic acids (FAs) are predominantly composed of aliphatic carbon and carboxylic groups.^33–35^ A high FA content is often indicative of an immature compost with a lower degree of humification.^33^ Generally, immature compost is distinguished by elevated FA levels and relatively low HA content, whereas mature compost exhibits lower FA concentrations and higher HA levels.^6^ During the composting process, FA concentrations tend to decrease while HA concentrations increase. In our study (Table 3), the HA/FA ratio was significantly high, ranging from 12.5 to 14.7 parts of HA for each part of FA, indicating that the compost produced had reached an advanced stage of maturation.

## 4 Conclusion

The composting of food waste resulted in the effective degradation of organic constituents, regardless of the season. Seasonal differences in air temperature and humidity contributed to the distinct degradation patterns observed since these environmental conditions directly influence microbial activity and the progress of the composting process. Both aeration and season influenced the composting process, with aeration promoting the degradation of TS (73.9%), C (75.1%), N (70.2%), and EE (97.4%) at 90 days in the summer. In contrast, during the winter, the absence of aeration also had significant effects, favoring reductions in TS (73.3%) and C (75.9%).

In terms of compost quality, aeration promoted the release of nutrients, including nitrogen and potassium, especially during the summer. However, the compost produced under nonaerated winter conditions reached a more advanced stage of maturation. While aeration benefits microbial activity, the lack of aeration can also result in high-quality compost with a higher degree of humification.

### 4.1 Plain Language Summary

Food waste composting, both with and without forced aeration, effectively reduces organic compounds and produces nutrient-rich biofertilizers, thereby contributing to the sustainable management of these waste materials.
